# Gender Differences among Elderly Patients with Primary Percutaneous Coronary Intervention

**DOI:** 10.14336/AD.2017.1129

**Published:** 2018-10-01

**Authors:** Binquan You, Bingbing Zhu, Xi Su, Feng Liu, Bingyin Wang

**Affiliations:** ^1^Departments of Cardiology, Suzhou Kowloon Hospital, School of Medicine Shanghai Jiaotong University, Suzhou, 215000, China; ^2^Department of Cardiology, Wuhan Asia Heart Hospital, Wuhan, 430000, China

**Keywords:** ST-segment elevation myocardial infarction, elderly female, primary percutaneous coronary intervention, major adverse cardiac events

## Abstract

Several epidemiological and clinical studies have shown that females with ST-segment elevation myocardial infarction (STEMI) have a higher mortality than males following primary percutaneous coronary intervention (PPCI). Many analyses of sex-based differences following STEMI have revealed conflicting results. Currently, more and more elderly patients with STEMI have undergone emergency interventional therapy. From January 2014 to December 2016, a total of 337 elderly patients with STEMI were enrolled in this study from two chest pain centers, and all patients underwent PPCI. Patients were divided into two groups: elderly females (n=117, mean age 73.4±9.6 years) and elderly males (n=220, mean age 71.7±8.6 years). The prevalence of diabetes was higher in females than in males (29.1% vs. 19.6%,P<0. 01). Typical ischemic chest pain was lower in females than in males (45.3% vs 57.3%, P<0.01). The number of nonsmokers was also significantly higher in females than in males (5.1% vs. 52.3%,P<0. 01). Serum creatinine (sCr) levels (87.6±17.4 umol/L vs 99.5±20.2 umol/L,P<0.01) and body mass index (23.8±2.7 vs 27.3±3.1, P<0.01) were lower in females than in males. The incidences of major adverse cardiac events (MACE) in-hospital showed no significantly difference (P>0.05) between the two groups. However, the cumulative MACE showed a significant difference between the two groups in the 12-month follow-up (16.8% in male vs 12.8% in female, P = 0.04). Our results suggest that the PPCI is safe and effective in elderly female STEMI patients. The cumulative MACE in females are not higher than in males. PPCI are helpful in elderly STEMI patients.

The influence of patient gender on outcomes of acute myocardial infarction (AMI) is controversial. Several studies indicate that a sex disparity exists in survival, with females having a higher mortality than males [1]. Data from a study of 45,852 patients in the COMMIT/CCS-2 study showed that compared with males of the same age, females had approximately a 50% higher mortality following hospital admission for ST-segment elevation myocardial infarction (STEMI) [2]. Studies have shown that coronary artery intervention in elderly patients with acute myocardial infarction, especially for women over 65 years of age, the incidence of cardiovascular events was significantly increased [3]. In a meta-analysis using data from 149 studies representing 18, 555 females and 49, 981 men, Pancholy *et al* found that in the unadjusted analyses, women were at a higher risk for in-hospital major adverse cardiac events (MACE) and a 1-year all-cause mortality compared with men [4]. An increased mortality in females with STEMI treated with percutaneous coronary intervention (PCI) was detected in this large meta-analysis, but is likely confounded by baseline cardiovascular risk factors and the differences in clinical profiles of male and female patients with STEMI. Wilkinson P et al suggested that the poorer prognosis for the American population of females with acute myocardial infarction was influenced by particularly high mortality among black females [5]. Many analyses of sex-based differences following acute coronary syndromes (ACS)-usually caused by STEMI, non-ST elevation myocardial infarction or unstable angina-have revealed conflicting results. Females with ACS generally present with more clustering of risk factors that may contribute to their higher risk of mortality. The China Patient-Centered Evaluative Assessment of Cardiac Events (PEACE) Retrospective Study indicated that females experienced a higher increase in hospitalization rates for STEMI in China between 2001 and 2011 and were less likely to receive evidence-based aggressive therapies, especially revascularization and reperfusion. In addition to efforts to improve quality of care generally, understanding the reasons for this sex disparity and addressing these differences in care should be a priority [6]. Several studies demonstrated that after an AMI, fewer females than males underwent cardiac catheterization. Data from the MITI study and the Cooperative Cardiovascular Project suggested that during acute treatment of myocardial infarction a somewhat less aggressive therapy was performed in females as compared with males [7], suggesting that intensive cardiovascular risk modification efforts in females may help to reduce this sex disparity. As the Framingham study’s investigators suggested differences in the characteristics of the study populations are probably important. Sex-related differences in the early and late outcome after elective PCI are likely related to the small, limited amount of data due to the lack of randomized clinical studies including and a lower representation of females in clinical trials.

Recent studies found that among patients with STEMI treated by PCI, females were associated with a higher 30-day mortality and complication rates compared with males. Following multivariate analysis, female gender was not a significant predictor of long-term death following STEMI [8]. China PEACE research showed that in the past decade in China, hospital admissions for STEMI have risen in these patients along with comorbidities and the intensity of testing and treatment. Quality of care has improved for some treatments, but important gaps persist, and in-hospital mortality has not decreased between 2001 and 2011[9].

In the present study, based on a series of Chinese patients who living in south china admitted for STEMI, we (1) analyzed the clinical characteristics of STEMI elderly patients who underwent Primary PCI (PPCI); (2) evaluated the safety and efficacy of PPCI in these patients; and (3) evaluated the impact of gender on mortality and complications and mainly focused on MACE and mortality during one-year follow-up.

## MATERIALS AND METHODS

### Patients and clinical features

1045 patients with STEMI were admitted to Suzhou Kowloon Hospital and Wuhan Asia Heart Hospital from January 1, 2015 to Sep 30, 2016. Among them, 337 patients were ≧ 65 years old and underwent PPCI. All patients were divided into two groups-females 117 (mean age 73.4±9.6 years, range 65-91 years), and males 220 (mean age 71.67±8.59 years, range 65-89 years). Patient data at each site were input into a computer database using a standardized electronic case report form then transferred to the database center through the internet by trained research doctors. The case report form included patient’s demographic characteristics, presenting symptoms, medical histories, in-hospital treatments, major outcomes, and discharged medications. This study was conducted in accordance with the World Medical Association Declaration of Helsinki then approved by the ethics committees at both hospitals. Patient information was encrypted prior to analysis. The AMI diagnostic criteria were based on the ESC Guidelines for the management of acute myocardial infarction in patients presenting with ST-segment elevation [10]. The inclusion criteria were: 1) onset of STEMI within 12 h, and 2) patient consent to PPCI. The exclusion criteria were: 1) onset of STEMI >12 h, 2) suspected aortic dissection, 3) remedial PCI after thrombolysis treatment, and 4) severe hepatorenal dysfunction.

### Operative procedures

Primary PCI: After hospital admission, patients received an18-lead electrocardiography (ECG) followed by ECG monitoring, oxygen inhalation, blood glucose, blood lipids, myocardial enzyme, and troponin tests as well as other related biochemical and routine tests. At the same time, patients took 300 mg of aspirin and 600 mg of clopidogrel (or Ticagrelor 180 mg) by chewing. The Judkins technique of coronary angiography was used in which a catheter was guided through the blood vessel to clear narrowed or blocked coronary arteries. Patients received 3000 and 8000 U heparin (100 u/kg) through a sheathing canal before and after angiography. A thrombus suction catheter was used to remove the thrombus if a thrombus shadow was visible under angiography after which a guide wire was passed through the culprit vascular lesions. When necessary, tirofiban was injected into coronary arteries with a dose of 10 µg/kg within 3min, followed by continuous intravenous (IV) administration at 0.15µg/(kg•min) by pumping for 24 h. Percutaneous transluminal coronary angioplasty with stent implantation or primary stenting was performed depending on the disease condition. The levels of myocardial enzymes and troponin, ECG, echocardiography, as well as hepatorenal function were reviewed after procedure. Patients continued to take 100 mg/day aspirin, 75 mg/day clopidogrel (or Ticagrelor 90 mg, bid) statins, β-receptor blockers, and hypoglycemic agents. Only culprit vessels were treated during PPCI, and other lesion vessels (if any) were treated by a second procedure 7-14 day after the PCI.

Diabetes mellitus was defined as a fasting plasma glucose concentration of ≧7.0mmol/L, of ≧11.0 mmol/L on a 75g, 2h oral glucose tolerance test, or the use of anti-diabetic treatment. Hypertension was defined as a history of a systolic blood pressure of ≥140 mmHg, a diastolic pressure of ≧90 mmHg, or the use of anti-hypertensive treatment (Note: On 11/14/2017, the American Heart Association published a new diagnostic criterion stating that hypertension is defined as SBP ≧130 mmHg, or DBP≧80 mmHg). Hyperlipidemia was defined as a fasting total blood cholesterol concentration of ≧220 mg/dl, a fasting triglyceride concentration of ≧150 mg/dl, or the use of anti-hyperlipidemic treatment.

**Table 1:** Baseline characteristics of the two groups.

Variable	Female group (n=117)	Male group (n=220)	P value
Age	73.4±9.6	71.7±8.6	0.15
BMI	23.8±2.7	27.3±3.1	< 0. 01
Smoking			
(%)Non-smoker	105 (89.7)	53 (24.1)	< 0. 01
Former-smoker	6 (5.1)	52 (23.6)	< 0. 01
Smoker	6 (5.1)	115 (52.3)	< 0. 01
Peripheral artery disease (%)	7 (6.0)	10 (4.6)	0.14
Dyslipidaemia (%)	78 (66.7)	142 (64.6)	0.13
Hypertension (%)	86 (73.5)	155 (70.5)	0.17
Diabetes mellitus (%)	34 (29.1)	43 (19.6)	< 0. 01
Atrial fibrillation (%)	11 (9.4)	19 (8.6)	0.15
History of stroke (%)	11 (9.4)	21 (9.6)	0.22
History of cardiac arrest (%)	2 (1.7)	5 (2.3)	0.12
Prior MI (%)	5 (4.3)	13 (5.9)	0.11
Prior PCI (%)	7 (6.0)	19 (8.64)	0.08
Prior CABG (%)	1 (0.86)	3 (1.36)	0.21
prior to admission (%)	29 (24.8)	65 (29.6)	0.06
Presenting symptoms			
Typical anginal chest pain (%)	53 (45.3)	126 (57.3)	< 0. 01
Atypical chest pain (%)	38 (32.5)	67 (30.5)	0.22
No chest pain (%)	26 (22.2)	27 (12.3)	< 0. 01
Killip class (preoperative, %)			
1	61 (52.1)	128 (58.2)	0.17
2	29 (24.8)	57 (25.9)	0.26
3	17 (14.5)	30 (13.6)	0.28
4	10 (8.6)	15 (6.8)	0.19
MI localization in ECG (%)			
anterior wall	47 (40.2)	101 (45.9)	0.11
inferior wall	40 (34.2)	86 (39.1)	0.10
Others	30 (25.6)	33 (15.0)	0.03
Troponin I on admission (ng/ml)	5.7±3.2	1.90±2.1	< 0. 01
sCr on admission (umol/L)	87.6 ± 17. 4	99.5 ± 20.2	< 0. 01
Blood glucose on admission (mmol/L)	7.2 ± 3.2	7.0 ± 2.9	0.25

BMI: Body mass index; sCr: Serum, creatinine; MI: myocardial infarction; PCI: percutaneous coronary intervention; CABG: Coronary artery bypass grafting.

Observed indexes were: (1) patient’s basic information, including age; Killip classification during myocardial infarction; history of coronary heart disease risk factors of smoking, hypertension, diabetes, hyperlipidemia and other conditions; old myocardial infarction (OMI); PCI history; history of stroke; renal dysfunction; and cardiopulmonary resuscitation situation before admission, (2) Angiography and interventional characteristics of the distribution of the infarct-related artery lesion count, thrombectomy, stenting, aortic balloon counter pulsation within (IABP) ratio, the success rate and contrast agent PCI operation, perspective time. (Note: The success of a PCI procedure was defined by angiographic, procedural, and clinical criteria, including postoperative vascular lesions to reduce the minimum diameter stenosis or less, and no significant clinical complications during hospitalization), (3) the clinical outcome of the situation, PCI 24 h after expert echocardiography measurement of left ventricular ejection fraction (LVEF); recorded interventions and clinical complications during hospitalization (stroke, heart failure, cardiogenic shock and hospital mortality), and (4) first medical contact-to-balloon time (FMC-to-B) and door-to-balloon time (D-to-B), average length of stay, average hospitalization and hospital mortality.

### Follow-up

All patients received follow-up 12 months after discharge. Follow-up was done on an outpatient clinic basis or through telephone contact. All clinical and imaging data were recorded in our database. MACE was defined as a composite of cardiovascular death, nonfatal MI and nonfatal stroke or hospitalization for heart failure. Patients were advised to repeat angiography once within a year of the procedure, but angiography was not done routinely for every patient. All deaths were confirmed by medical records or telephone interviews with patients’ families, and death was regarded as being cardiovascular in origin unless obvious non-cardiovascular causes were identified.

### Statistical analysis

Statistical analysis was carried out with the software package SPSS, and the mean values and frequencies of various risk factors (variables) were studied in the groups. All variables were presented as mean ± standard deviation (SD). Comparison between groups was performed using Student’s t-test (unpaired). Proportions were compared using χ^2^ analysis. Multivariate logistic regression was used to determine the correlation of baseline characteristics to SD. Differences with P < 0.05 were considered statistically significant.

## RESULTS

### Baseline Characteristics

The baseline clinical characteristics of the two groups are listed in Table 1. The female group of 117 cases had an average age of 73.4±9.6 years; the male group of 220 cases had an average age of 71.7±8.6 years. Body mass index (BMI) and non-smoking rates were significantly different between the two groups (P<0.01).The proportion of diabetes in the females group was significantly higher than in the male group (29.1% vs. 19.6%, P<0.01), but there was no significant difference in blood glucose at admission (P>0.05).The peripheral vascular, hypertension, atrial fibrillation, stroke, myocardial infarction, previous surgery PCI, CABG and the rate of abnormal lipid metabolism showed no significant difference between the two groups.

Typical coronary ischemic chest pain was significantly lower in elderly female patients than in elderly male patients (45.3% vs. 57.3%, P<0.01). The position of myocardial infarction judged by electrocardiogram showed no difference. There was no difference of Killip class on admission between the two groups (P>0.05). Troponin I level on admission of the female group was significantly higher than the male group (5.72 ± 3.24 vs. 1.90 ± 2.12, P<0.01), while the male group with poor renal function and the male serum creatinine level group was significantly higher than the female group (99.5 ± 20.2 vs. 87.6 ± 17.4, P<0.01).

### Angiographic data and the results of primary PCI in the two groups

Characteristics of coronary artery lesions in the two groups are listed in Table 2. Infarct-related coronary artery, number of implanted stents, stent diameter and stent length were not significantly different between the two groups (p>0.05). Operative time(min), successful PCI (%), and final flow in the infarct-related artery (TIMI) showed no significant differences between the two groups (P>0.05).

The rate of emergency calls was low in elderly female patients than in elderly male patients (11.97% vs. 19.09%, p<0.01). D-to-B time of two groups showed no significant difference, but symptom-to-balloon time in the female group was significantly longer than the time in male group (460.8 ± 81.9 min vs. 400.4 ± 75. 5, P<0.01). There was no significant difference in the distribution of offenders of the two groups (P>0.05).

### Cumulative MACE during one-year follow-up in two groups

Cumulative MACE during one-year follow-up in the two groups were listed in Table 3.

There were no significant differences in reinfarction, stroke, postoperative bleeding and MACE in-hospital between two groups. No significant differences were found between the two groups. However, the rate of MACE showed a significant difference in the 12month follow-up (16.8% in males vs. 12.8% in females, P = 0.04).

**Table 2:** Comparison of characteristics of coronary artery lesions in the two groups.

Variable	Women (n= 117)	Men (n= 220)	P value
Infarct-related coronary artery (%)			
Left main coronary artery	1 (0.85)	3 (1.36)	0.18
Left anterior descending artery	47 (40.2)	101 (45.9)	0.09
Circumflex artery	25 (21.4)	41 (18.6)	0.08
Right coronary artery	44 (37.6)	75 (34.1)	0.14
Emergency Call (%)	14 (11.97)	43 (19.09)	< 0. 01
S-to-B (min)	460.8±81.9	400.4±75. 5	< 0. 01
FMC-to-B (min)	109	116	0.26
Door-to-balloon time (min)	74.38±19.76	72.65±20.76	0.20
Thrombuster (%)	7(5.98)	18 (8.2)	0.07
IABP (%)	9(7.7)	17 (7.7)	0.31
Temporary pacing (%)	14(12.0)	21 (9.6)	0.19
Medical therapy (%)			
Aspirin	115 (98.3)	218 (99.1)	0.29
Clopidogrel/Tegreino	113 (96.6)	215 (97.7)	0.19
β-blocks	85 (72.6)	162 (73.6)	0.21
ACEI/ARB	98 (83.8)	186 (84.5)	0.26
Statins	107 (91.5)	202 (91.8)	0.29
Severity of CAD (%)			
one-vessel	36 (30.8)	67 (30.5)	0.37
two-vessel	58 (49.6)	121 (55.0)	0.06
three-vessel	23 (19.7)	32 (14.6)	0.04
Preoperative TIMI 0-1 (%)	102 (87.1)	201 (91.4)	0.09
Postoperative TIMI (%)			
3	96 (82.1)	184 (83.6)	0.19
2	15 (12.8)	22 (10.0)	0.16
0-1	6 (5.1)	14 (6.4)	0.17
Operative time(min)	60.2 ± 13.6	56. 3±11.8	0.10
Successful PCI (%)	105 (89.7)	201 (91.4)	0.22
Stent number	1.38±0.76	1.36±0.90	0.25
Stent diameter (mm)	2.89±0.79	3.35±0.94	0.17
Stent length (mm)	31.51±3.08	33.45±3.34	0.13
Average length of hospital stays (d)	10.73±3.22	9.65±2.86	0.10

TIMI: Final flow in the infarct-related artery; S-to-B:Symptom to balloon time; FMC to B:first contact time to balloon time; D-to-B:Door-to-balloon time; CAD: Coronary artery disease.

### Survival

**Figure 1.**
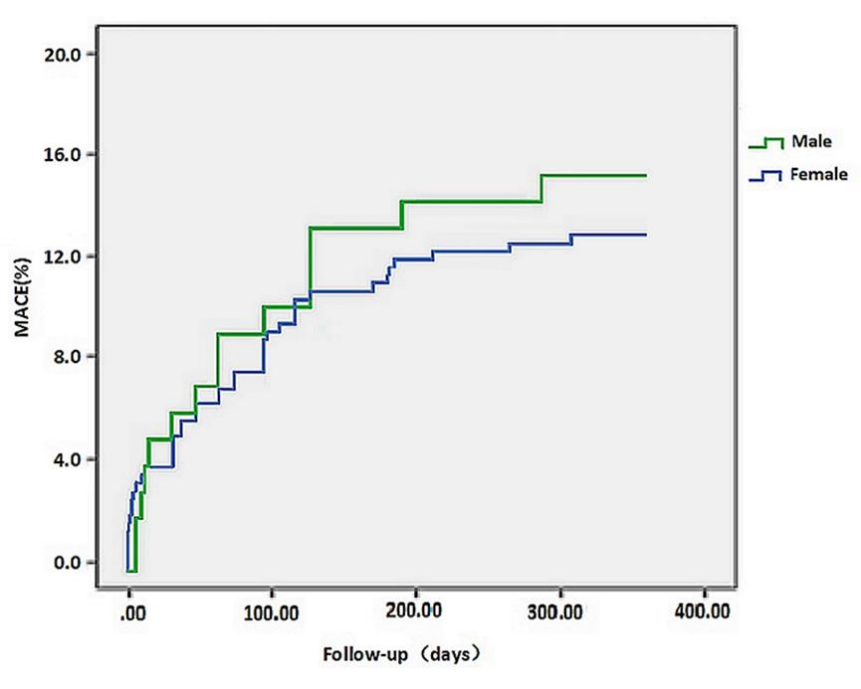
The Kaplan-Meier survival curves of Cumulative MACE during one-year follow-up in two groups.

Kaplan-Meier analysis was shown in Figure 1. The rate of MACE showed no significant difference between the two groups at in-hospital and one-month follow-up. However, there was a significant difference between the two groups with respect to one-year MACE during the follow-up period.

## DISCUSSION

The main findings of the present study of patients with STEMI undergoing PCI are: (1) The incidence of MACE was significantly lower in elderly females than in older males in patients with STEMI after one year of PCI treatment while the cardiac death was no significant difference between females and males; (2) SCr levels and renal function, BMI and smoking in elderly patients with STEMI at admission were important factors affecting mortality and MACE at 30 days and one days of PCI.

Timely reperfusion, effective mechanical recanalization of the obstructed coronary arteries, and aggressive antiplatelet therapy have dramatically improved the prognosis of patients with acute STEMI [11]. The influence of patient sex on outcomes of PCI is controversial. Elderly patients with STEMI are at high risk for complications and early mortality. Mortality in female patients with STEMI undergoing primary angioplasty (PCI) is higher than in males. In a large meta-analysis of 11 randomized ACS trials it was shown that sex-based differences in 30-day mortality among patients with various manifestations of ACS are largely explained by clinical differences at presentation and the severity of angiographically documented disease [12]

In this study, we observed that 30-days after PCI there were no significant differences in MACE and cardiac death (CD) between elderly females and males with STEMI. While,12 months long-term CD (12 months follow-up) in elderly female patients was slightly lower than the elderly male (8.6% vs 10.5%, P=0.05), MACE was also lower in females than in males (12.8% vs 16.8%, P=0.01). This is different from past literature reports. Why does this difference occur? Which factors affect/cause such a change? Looking at the data we collected, we suggested that the following factors may be involved.

### sCr levels and renal function

In this study, we observed that sCr (serum, creatinine) at admission was significantly lower in elderly females treated with PCI than in males. Claussen and his colleagues studied the risk profiles at presentation and early mortality in elderly (≧80 years) versus younger (<80 years) STEMI patients. High levels of serum cholesterol and creatinine were important risk factors for early mortality in elderly patients [13]. Chen et al. also found serum Cr concentration on admission was a strong predictor for in-hospital mortality among Chinese acute STEMI patients especially in the young and the female [14]. Gevaert et al. examined gender differences in the prevalence and prognostic performance of renal dysfunction at admission in this setting. Female gender was independently associated with renal dysfunction at admission in primary PCI treated patients. Renal dysfunction was equally associated with higher in-hospital mortality in both males and females [15]

**Table 3:** Cumulative MACE during one - year follow-up in two groups.

Variable	Female (n=117)	Male (n=220)	P value
**In hospital** (%)			
CHF need treatment	23 (19.7)	41 (18.6)	0.15
Bleeding complication	19 (16.2)	30 (13.6)	0.02
Severe arrhythmia	22 (18.8)	47 (21.4)	0.03
Myocardial infarction	2 (1.71)	4 (1.8)	0.37
Stroke	1 (0.86)	1 (0.46)	0.48
Cardiogenic shock	7 (5.13)	10 (4.55)	0.27
In-hospital death	3 (2.56)	6 (2.73)	0.37
MACE	6 (5.13)	11 (5.00)	0.60
**One month (%)**			
Myocardial infarction	3 (2.56)	6 (2.73)	0.07
Cardiac death	5 (4.27)	10 (4.55)	0.15
Stroke	0 (0.00)	1 (0.46)	0.18
MACE	8 (6.84)	17 (7.73)	0.11
**In 12 months**			
Myocardial infarction	4 (3.42)	10 (4.55)	0.09
Cardiac death	10 (8.55)	23 (10.45)	0.11
Stroke	2 (1.71)	4 (1.82)	0.58
MACE	15 (12.82)	37 (16.82)	0.04

CHF: Congestive heart failure, major adverse cardiac event

### BMI and MACE in STEMI patients

In the present study, we observed that MBI in elderly females was significantly lower than in elderly males (23.8 vs. 27.3). The Body Mass Index (BMI) is a major predictor for a wide range of chronic diseases and injuries including cardiovascular disease (CVD) and is still the most frequently studied anthropometric index on outcomes of patients undergoing percutaneous coronary angioplasty (PCI). In a large case-control study involving >12?000 cases of myocardial infarction (MI) and 14?000 controls of varying ethnicity from 52 countries, Yusuf *et al*. found that BMI was positively and linearly associated with MI [16]. Joyce et al reported about a large cohort of patients with first STEMI treated with primary percutaneous coronary intervention who were followed for 5.2 years to assess the effects of myocardial function and BMI on outcome defined as all-cause mortality. They found that obese patients had greater adverse left ventricular (LV) remodeling and a significantly more impaired LV global longitudinal strain (GLS) after STEMI compared with those with normal BMI, amid similar infarct characteristics [17]. In a large recent registry study which looked at all-cause mortality according to BMI in 64436 patients with acute coronary syndrome, a similar inverse relationship between BMI and mortality was maintained regardless of whether patients were treated with an invasive strategy or medical therapy alone [18].

### Smoking and myocardial infarction

Ample evidence shows the harmful nature of smoking. Cigarette smoking is one of the most important risk factors for acute myocardial infarction (MI) worldwide, and the subsequent morbidity and mortality. In the present study, we observed that PCI-treated smokers in the elderly female patient group were significantly less likely to be smokers than males (5.1%, vs, 52.3%, P<0.01).

In a dose- and duration-dependent fashion, both active and passive exposure to cigarette smoking is associated with greater risk for future cardiovascular events [19]. Steele found cigarette smoking is associated with a fivefold increased risk of STEMI [20]. On a general population level, in a recent pooled analysis of cohort studies including more than 500,000 patients, smokers had a twofold hazard of acute coronary events, with an estimated earlier risk for cardiovascular death of 5.5 years [21]. The low smoking rate in the female group may be an important cause of lower MACE levels than the male group in our study.

### Chest pain in elderly patients with STEMI

Elderly adults with ACS are at risk for higher mortality rates. Early recognition of symptoms can improve patient outcomes. Chest pain or discomfort is regarded as the hallmark symptom of ACS, and its absence is regarded as “atypical” presentation. Elderly adults reporting an absence of chest pain on arrival are twice as likely to die compared with elderly adults with chest pain. Compared with elderly males in our study, we observed that elderly females had less typical chest pain (45.3% vs 57.3%, P<0.01) and a greater lack of chest pain (22.2% vs 12.3%, P< 0.01).

Canto *et al*. also reported that the absence of chest pain or discomfort with ACS was noted more commonly in females than in males in both the cumulative summary from large cohort studies (37% vs 27%) and the single-center and small reports or interviews (30% vs. 17%). They noted that males are more likely to present with chest pain whereas females are more likely to present with nausea. Elderly patients who present to the emergency department with ACS and a chief complaint other than chest pain are often misdiagnosed and under-treated and have higher in-hospital mortality rates than adults aged younger than 65 years with chest pain [22].
